# An individualized nomogram for predicting risk of sepsis in patients with pyogenic liver abscesses: a 10 years retrospective analysis

**DOI:** 10.3389/fmed.2025.1555656

**Published:** 2025-07-09

**Authors:** Tiezhao Zhang, Xidong Cao, Liyong Zhang, Jinhua Cui, Jian Li, Ziyu Bai, Aijun Yu

**Affiliations:** The First Department of General Surgery, Affiliated Hospital of Chengde Medical University, Chengde, Hebei, China

**Keywords:** nomogram, prediction, prognosis, pyogenic liver abscesses, sepsis

## Abstract

**Introduction:**

The incidence of pyogenic liver abscess (PLA) has been increasing. A poor prognosis and high mortality rate are observed when PLA progresses to sepsis. Thus, it is crucial to identify patients at high risk of sepsis early and develop personalized treatment plans to reduce the disease burden of patients with liver abscesses. However, a substantial research gap exists in the prediction of sepsis in patients with PLA.

**Methods:**

A retrospective study involving 490 patients with PLA was conducted. In chronological order, patients treated from August 2014 to September 2021 were employed as the training cohort (*n* = 341), and patients treated from October 2021 to July 2024 were employed as the validation cohort (*n* = 149). The occurrence of sepsis in patients with liver abscesses was defined as the outcome. The Chi-square test or Fisher’s exact test was used to test categorical variables, whereas the Student’s *t*-test was employed to evaluate continuous variables. Independent risk factors associated with sepsis in the training cohort were identified using multivariate logistic regression analysis. A nomogram was developed and validated using an independent cohort. Model performance was systematically evaluated through three analytical approaches. Receiver operating characteristic (ROC) curves were generated for both the training and validation cohorts to assess discrimination accuracy. Calibration curves were constructed to quantify the agreement between predicted and observed outcomes. Decision curve analysis (DCA) was conducted to determine the clinical utility threshold where the nomogram’s net benefit surpassed empirical treatment strategies across both cohorts.

**Results:**

A total of 108 (22%) patients with PLA were complicated with sepsis. In patients with liver abscesses, independent risk factors for sepsis, including white blood cell count, international normalized ratio (INR), presence of gas, and sequential (sepsis-related) organ failure assessment (SOFA) score, were identified through multivariate logistic regression analysis. For the training and validation cohorts, the area under the curve (AUC) values of the nomogram were 0.880 (95% CI: 0.832–0.929) and 0.901 (95% CI: 0.839–0.964), respectively, showed that the newly established nomogram exhibited superior predictive performance and clinical utility. The Hosmer–Lemeshow test (χ^2^ = 8.60, *P* = 0.377) suggests good fit. The calibration curve showed good consistency, and the DCA decision curve showed that the model was clinically effective.

**Conclusion:**

A model with four clinical features was developed to predict the risk of sepsis in patients with liver abscesses. The model exhibited good predictive ability during time verification.

## 1 Introduction

Pathogenic bacteria cause pyogenic liver abscess (PLA), a suppurative liver lesion, through hematogenous, biliary, or intestinal routes. PLA is the most common abscess in the abdominal cavity, affecting men more than women (1, 2). PLA is associated with high morbidity and mortality rates. The PLA incidence rate in Western countries ranges from 1.07 to 3.59 per 100,000 hospitalized patients, whereas it can be as high as 17.59 cases per 100,000 individuals in Eastern countries, with a mortality rate ranging from 2.8% to 10.8% (3). Recently, in Asia, *Klebsiella pneumoniae* (KP) has surpassed *Escherichia coli* (*E. coli*) as the most significant PLA pathogen (4). However, it is easy to miss and misdiagnose during the initial diagnosis because of the extended cycle of pathogen culture, leading to prolonged diagnosis and treatment, as well as poor prognosis.

Sepsis is considered a serious complication of infectious diseases. Multiple organ dysfunction, septic shock, and even death can be caused by organ dysfunction resulting from the host’s response to infection (5, 6). Identifying the risk factors for sepsis in patients with liver abscesses is a top priority to mitigate the risk. Studies on risk factors related to sepsis in patients with liver abscesses have been limited, and the results are not entirely consistent. KP infection, the presence of tumors, liver failure, and septic shock have been identified in previous studies as independent risk factors for mortality in these patients (7). Comprehending these risk factors can enhance awareness among doctors and patients and prompt additional interventions. However, a deeper understanding of precision medicine is required because of the rapid development of medical care, enabling the evaluation of individual patients and the tailoring of treatment approaches.

A clinical prediction model is a statistical multivariate mathematical model that processes relevant predictive indicators. Currently, there is a significant lack of risk prediction models for sepsis in patients with liver abscesses. This study aimed to identify independent risk factors that may contribute to the development of sepsis in patients with liver abscesses, create a simple and intuitive personalized model, and validate its performance. The nomogram offers an intuitive prediction of the clinical risk of disease progression (8). Informed diagnoses and treatment plans can be made by doctors with its assistance, allowing for timely interventions to reduce the chances of sepsis in patients with liver abscesses and to mitigate the associated burden on patients and society.

## 2 Materials and methods

### 2.1 Patients

A retrospective analysis of the electronic medical records of patients primarily diagnosed with liver abscesses and discharged from the Affiliated Hospital of Chengde Medical University between 1 August 2014, and 30 July 2024, was conducted. Subsequently, the electronic medical records were reviewed by two researchers according to the diagnostic criteria for PLA. This study was conducted in accordance with the Declaration of Helsinki and was approved by the Ethics Committee of the Affiliated Hospital of Chengde Medical University (No. CYFYLL2022507). The requirement for informed consent was waived because of the retrospective nature of this study. The inclusion criteria for PLA are as follows: (1) clinical manifestations, including fever, chills, jaundice, and upper abdominal discomfort; (2) confirmation of liver abscess or space-occupying lesions through abdominal imaging examination (ultrasound/CT/MRI); (3) positive pus culture obtained from peripheral blood or lesion puncture or effective anti-infection treatment; and (4) diagnosis of liver abscess confirmed through percutaneous liver puncture or surgical treatment. Based on (1) and (2), any one or more of (3) and (4) can be diagnosed as PLA. Exclusion criteria: (1) age below 18 years; (2) incomplete clinical data or significant lack of laboratory examination data; and (3) conditions such as liver liquefaction infarction, hepatic echinococcosis, amebic liver abscess, or tuberculous liver abscess. Based on the Third International Consensus on Sepsis and Septic Shock in 2016 (9), sepsis can be diagnosed if any of the following conditions are satisfied after an infection: (1) sequential (sepsis-related) organ failure assessment (SOFA) ≥ 2 points; (2) the baseline SOFA score can be assumed to be zero in patients not known to have preexisting organ dysfunction, and a cumulative increase in SOFA score ≥ 2 points. Based on the presence or absence of sepsis complications, patients were classified into sepsis and non-sepsis groups. This study included 490 patients with liver abscesses. The training cohort (*n* = 341) comprised patients treated between August 2014 and September 2021, whereas the validation cohort (*n* = 149) comprised patients treated from October 2021 to July 2024 for model evaluation.

### 2.2 Data collection

Demographic data and clinical information, such as gender and age, were collected from electronic medical records. The electronic medical records provided data on various aspects, including basic diseases (hepatitis, cirrhosis, biliary tract disease, fatty liver, malignant tumor, hypertension, type 2 diabetes, hyperuricemia, and abdominal surgery), source of infection (biliary tract, intestinal, abdominal, other, or unknown), symptoms and signs (fever, chills, poor appetite, dyspnea, neurological symptoms, nausea and vomiting, abdominal pain, fatigue, and liver percussion pain), imaging results (abscess location: left lobe, right lobe, bilateral lobe, caudate lobe; presence of gas), bacterial species (KP, *E. coli*, and other bacteria), complications (pleural effusion, peritoneal effusion, pulmonary infection, perihepatic abscess, infective shock, KPLA invasion syndrome, and sepsis), treatment approaches (simple antibiotic treatment, antibiotic + puncture drainage, and surgical incision and drainage), and laboratory examination findings [white blood cell (WBC) count, international normalized ratio (INR), neutrophil count (NEC), Platelet count (PLT), C-reactive protein (CRP), blood glucose, alkaline phosphatase, fibrinogen, number and size of abscesses, and SOFA score). For patients who underwent multiple laboratory tests during hospitalization, only the initial test results were included.

### 2.3 Statistical analysis

IBM SPSS Statistics 25 software was used to perform statistical analysis. The cohort data were categorized into a training cohort (*n* = 341) and a validation cohort (*n* = 149). Variables with more than 20% missing values (procalcitonin, interleukin, and lactic acid) were excluded from the analysis. Missing data were addressed using the fully conditional specification multiple imputation method for variables with missingness rates < 20% (fibrinogen, CRP, and alkaline phosphatase). Ten imputation cycles were performed, with algorithm specifications tailored to variable types: predictive mean matching (PMM) for continuous variables, logistic regression for binary categorical variables, and proportional odds models for ordinal categorical variables. *T*-tests or Wilcoxon rank-sum tests were used to conduct group comparisons of continuous variables. Categorical variables are presented as frequencies (percentages), whereas continuous variables are expressed as mean ± SD or median (P25 and P75). Initially, univariate analysis was conducted to identify factors associated with sepsis in patients with liver abscesses. In the multivariate analysis, factors with a *P* < 0.05 were included. Multivariable logistic regression analysis utilizing bidirectional stepwise selection was conducted to identify independent clinical predictors. The variable selection process employed entry and removal probability thresholds of *P* < 0.10 and *P* > 0.10, respectively. A two-sided *P* < 0.05 was considered statistically significant.

Statistical analysis was performed using R software (Version 4.3.1). Based on the findings of multiple logistic regression analysis, a prediction model for sepsis in patients with liver abscesses was developed. To evaluate the risk of sepsis, the RMS package was employed to create a nomogram. The agreement between the observed and predicted results was assessed using the Hosmer–Lemeshow goodness-of-fit test. The R glmnet and ROCR packages were employed to plot the receiver operating characteristic (ROC) curves for each model. To validate the model, bootstrap resampling with 1,000 iterations was performed, and the area under the receiver operating characteristic (AUROC) curve was computed as a measure of discrimination. The validation cohort was used to validate the model, and the AUROC and calibration curves were employed to evaluate the discriminative ability and predictive accuracy of the model. The net clinical benefit of the model was evaluated using decision curve analysis (DCA).

## 3 Results

### 3.1 Patient characteristics

**FIGURE 1:**
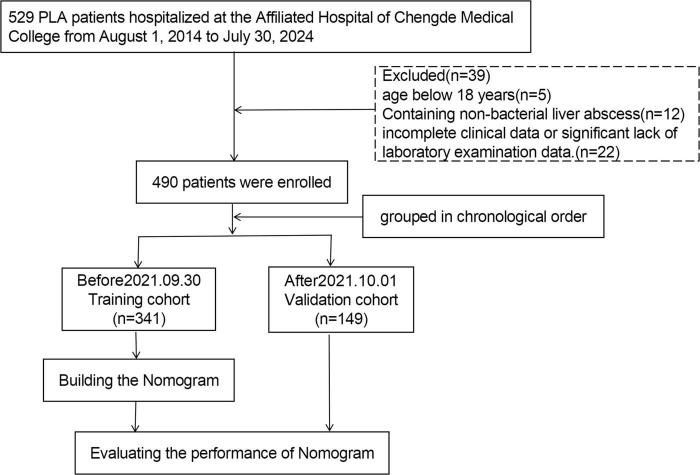
Flowchart of study patients.

A total of 490 patients, comprising 302 males and 188 females, were included in this study. Based on the chronological order of treatment, the patients were categorized into a training cohort (*n* = 341) and a validation cohort (*n* = 149) (Figure 1). Table 1 presents the clinical characteristics of the patients in the training and validation cohorts. No significant differences in the distribution of features were noted between the two cohorts (*P* > 0.05).

**TABLE 1:** Baseline characteristics of patients with liver abscess in the training and validation cohorts.

Variables	Total	Training cohort	Validation cohort	*P*-value
*N*	490	341	149	
Age	59.60 ± 13.47	59.04 ± 13.25	60.87 ± 13.94	0.169
Sex				0.114
Female	188 (38.4)	123 (36.1)	65 (43.6)	
Male	302 (61.6)	218 (63.9)	84 (56.4)	
Hepatitis				
Yes	20 (4.1)	16 (4.7)	4 (2.7)	0.302
No	470 (95.9)	325 (95.3)	145 (97.3)	
Hepatic cirrhosis				0.470
Yes	10 (2.0)	8 (2.3)	2 (1.3)	
No	480 (98.0)	333 (97.7)	147 (98.7)	
Biliary tract diseases				0.583
Yes	130 (26.5)	88 (25.8)	42 (28.2)	
No	360 (73.5)	253 (74.2)	107 (71.8)	
Fatty liver				0.977
Yes	10 (2.0)	7 (2.1)	3 (2)	
No	480 (98.0)	334 (97.9)	146 (98)	
Cancer				0.675
Yes	57 (11.7)	41 (12.1)	16 (10.7)	
No	432 (88.3)	299 (87.9)	133 (89.3)	
Hypertension				0.079
Yes	138 (28.2)	88 (25.8)	50 (33.6)	
No	352 (71.8)	253 (74.2)	99 (66.4)	
Type 2 diabetes mellitus				0.117
Yes	185 (37.8)	121 (35.5)	64 (43)	
No	305 (62.2)	220 (64.5)	85 (57)	
Hyperuricemia				0.065
Yes	31 (6.3)	17 (5)	14 (9.4)	
No	459 (93.7)	324 (95)	135 (90.6)	
Abdominal surgery				0.110
Yes	73 (14.9)	45 (13.2)	28 (18.8)	
No	417 (85.1)	296 (86.8)	121 (81.2)	
Source of infection				0.787
Biliary tract source	121 (24.8)	35 (23.5)	86 (25.4)	
Intestinal source	7 (1.4)	2 (1.3)	5 (1.5)	
Abdominal source	21 (4.3)	9 (6)	12 (3.5)	
Other source	4 (0.8)	1 (0.7)	3 (0.9)	
Unknown source	335 (68.6)	102 (68.5)	233 (68.7)	
Temperature (°C)	38.72 ± 1.96	38.78 ± 2.18	38.57 ± 1.29	0.131
Shiver				0.219
Yes	231 (47.1)	167 (49)	64 (43)	
No	259 (52.9)	174 (51)	85 (57)	
Poor appetite				0.104
Yes	249 (50.8)	165 (48.4)	84 (56.4)	
No	241 (49.2)	176 (51.6)	65 (43.6)	
Dyspnea				0.140
Yes	28 (5.7)	16 (4.7)	12 (8.1)	
No	462 (94.3)	325 (95.3)	137 (91.9)	
Nervous system symptoms				0.116
Yes	22 (4.5)	12 (3.5)	10 (6.7)	
No	468 (95.5)	329 (96.5)	139 (93.3)	
Nausea and vomiting				0.309
Yes	46 (9.4)	29 (8.6)	17 (11.5)	
No	441 (90.6)	310 (91.4)	131 (88.5)	
Abdominal pain				0.148
Yes	102 (20.8)	65 (19.1)	37 (24.8)	
No	388 (79.2)	276 (80.9)	112 (75.2)	
Fatigue				0.372
Yes	47 (9.6)	30 (8.8)	17 (11.4)	
No	442 (90.4)	310 (91.2)	132 (88.6)	
Liver percussion pain				0.058
Yes	127 (26.0)	97 (28.4)	30 (20.3)	
No	362 (74.0)	244 (71.6)	118 (79.7)	
Abscess location				0.107
Left lobe	109 (22.3)	83 (24.4)	26 (17.6)	
Right lobe	287 (58.8)	199 (58.5)	88 (59.5)	
Left and right lobe	89 (18.2)	55 (16.2)	34 (23)	
Caudate lobe	3 (0.6)	3 (0.9)	0 (0.0)	
Gas forming				0.610
Yes	57 (11.6)	38 (11.1)	19 (12.8)	
No	433 (88.4)	303 (88.9)	130 (87.2)	
Bacterial species				0.760
*Klebsiella pneumoniae*	440 (89.8)	306 (89.7)	134 (89.9)	
*Escherichia coli*	24 (4.9)	18 (5.3)	6 (4)	
Other bacteria	26 (5.3)	17 (5.0)	9 (6)	
Hydrothorax				
Yes	82 (16.7)	51 (15.0)	31 (20.8)	0.111
No	408 (83.3)	290 (85.0)	118 (79.2)	
Seroperitoneum				0.157
Yes	34 (6.9)	20 (5.9)	14 (9.4)	
No	456 (93.1)	321 (94.1)	135 (90.6)	
Pulmonary infection				0.125
Yes	56 (11.4)	34 (10.0)	22 (14.8)	
No	434 (88.6)	307 (90.0)	127 (85.2)	
Perihepatic abscess				0.294
Yes	6 (1.2)	3 (0.9)	3 (2.0)	
No	484 (98.8)	338 (99.1)	146 (98.0)	
Infective shock				0.599
Yes	60 (12.2)	40 (11.7)	20 (13.4)	
No	430 (87.8)	301 (88.3)	129 (86.6)	
MODS				0.164
Yes	15 (3.1)	8 (2.3)	7 (4.7)	
No	475 (96.9)	333 (97.7)	142 (95.3)	
*Klebsiella pneumoniae* liver abscess invasion syndrome				0.349
Yes	2 (0.4)	2 (0.6)	0 (0)	
No	488 (99.6)	339 (99.4)	149 (100)	
ICU admission				0.304
Yes	34 (6.9)	21 (6.2)	13 (8.7)	
No	456 (93.1)	320 (93.8)	136 (91.3)	
Treatment				0.136
Antibiotics	223 (45.5)	165 (48.4)	58 (38.9)	
Antibiotics and drainage	265 (54.1)	175 (51.3)	90 (60.4)	
Operative drainage	2 (0.4)	1 (0.3)	1 (0.7)	
WBC (×10^9^)	8.97 (6.5, 12.23)	9.12 (6.36, 12)	8.88 (6.97, 12.5)	0.510
Neutrophils ratio (%)	75.45 ± 12.84	74.48 ± 12.98	75.69 ± 12.25	0.501
Hb (g/L)	113.15 ± 21.76	113.81 ± 20.78	111.65 ± 23.86	0.559
PLT (×10^9^)	239.5 (140, 341)	248 (141, 353.5)	227 (135.5, 333)	0.316
CRP (mg/L)	105 (64.93, 155.51)	100.67 (62.88, 151.34)	116 (72.54, 163.55)	0.175
Blood glucose (mmol/L)	7.64 (5.84, 11.3)	7.78 (5.85, 11.54)	7.33 (5.79, 10.57)	0.385
INR	1.147 ± 0.188	1.138 ± 0.174	1.166 ± 0.216	0.266
ALP (U/L)	162.38 (115.13, 233.29)	165 (120.5, 237.5)	159 (106.42, 217.51)	0.217
Fibrinogen (g/L)	5.56 (4.59, 6.49)	5.33 (4.58, 6.47)	5.82 (4.87, 6.55)	0.140
Number of abscesses				0.222
Single action	267 (54.5)	192 (56.3)	75 (50.3)	
Multiple	233 (45.5)	149 (43.7)	74 (49.7)	
Diameter (mm)	62 (48.0, 83.0)	63 (49.5, 83.0)	58 (34.3, 83.5)	0.151
SOFA score	0 (0, 0)	0 (0, 1)	0 (0, 1)	0.169

Data are expressed as mean ± SD, number (percentage), or median (25th/75th percentile). WBC, white blood cell; Hb, hemoglobin; PLT, platelet; CRP, C-reaction protein; INR, international normalized ratio; ALP, alkaline phosphatase; SOFA, sequential organ failure assessment.

### 3.2 Univariate and multivariate logistic regression analyses

In the univariate analysis, statistically significant factors associated with sepsis in patients with liver abscesses included abdominal pain (*P* = 0.003), fatigue (*P* = 0.013), WBC (*P* < 0.001), NEC (*P* < 0.001), INR (*P* = 0.005), gas-forming (*P* = 0.034), poor appetite (*P* = 0.045), SOFA score (*P* < 0.001), and infective shock (*P* = 0.007). In the multivariate logistic regression analysis, independent risk factors for sepsis in patients with liver abscess were identified as WBC (*P* < 0.001), INR (*P* = 0.025), gas formation (*P* = 0.030), and SOFA score (*P* < 0.001) (Table 2).

**TABLE 2:** Univariate and multivariate logistic regression analyses.

	Univariate analysis		Multivariate analysis	
Variables	HR (95% CI)	*P*-value	HR (95% CI)	*P*-value
Abdominal pain	2.748 (1.369, 5.519)	0.003		
Fatigue	3.285 (1.222, 8.830)	0.013		
WBC	1.36 (1.253, 1.476)	<0.001	1.33 (1.214, 1.458)	<0.001
NEC	1.058 (1.031, 1.085)	<0.001		
INR	9.064 (2.068, 39.730)	0.005	7.650 (1.292, 45.306)	0.025
Gas forming	4.795 (2.384, 9.464)	0.034	2.882 (1.107, 7.500)	0.030
Poor appetite	1.687 (1.009, 2.821)	0.045		
SOFA	4.363 (2.560, 7.436)	<0.001	3.333 (1.75, 6.349)	<0.001
Infective shock	2.776 (1.292, 5.963)	0.007		

### 3.3 Construction of the nomogram prediction model

**FIGURE 2:**
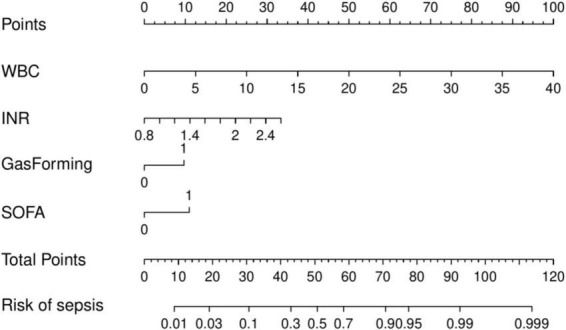
Nomogram for predicting the risk of sepsis in patients with liver abscess.

Multivariate logistic regression analysis revealed that WBC, INR, gas formation, and SOFA score were independent risk factors for sepsis development in patients with liver abscesses. A prediction model was developed, and a nomogram (Figure 2) was created to visually represent the model based on these results. The nomogram enables the estimation of sepsis risk in patients with liver abscesses by summing the points assigned to each of the four indicators on their respective scales. The total score corresponds to the probability of sepsis risk.

### 3.4 Validation of the nomogram

**FIGURE 3:**
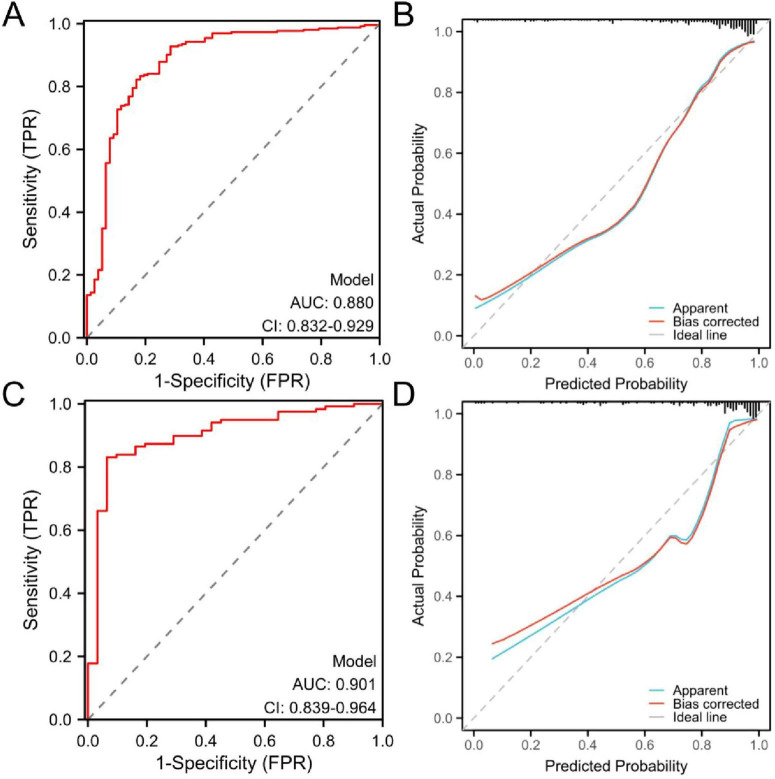
Receiver operating characteristic (ROC) and calibration curves of the prognostic nomogram model. **(A)** ROC curves of the training cohort and **(B)** calibration curve of the training cohort. **(C)** ROC curves of the validation cohort and **(D)** calibration curve of the validation cohort. ROC, receiver operating characteristic; AUC, area under the ROC curve.

The area under the curve (AUC) value, calibration curve were used to assess the predictive performance of the established model. In the training cohort (Figure 3A) and the validation cohort (Figure 3C), the AUC values of the prognostic model were 0.880 (95% CI: 0.832–0.929) and 0.901 (95% CI: 0.839–0.964), respectively. The findings demonstrated that the AUC values of the model were higher than 0.80 in the training and validation cohorts, indicating good discriminative ability.

In both the training cohort (Figure 3B) and the validation cohort (Figure 3D), the calibration curve demonstrated an acceptable level of agreement between the predicted and observed probabilities of the model. Hosmer–Lemeshow showed good agreement between the predicted and observed results (χ^2^ = 8.60, *P* = 0.377).

**FIGURE 4:**
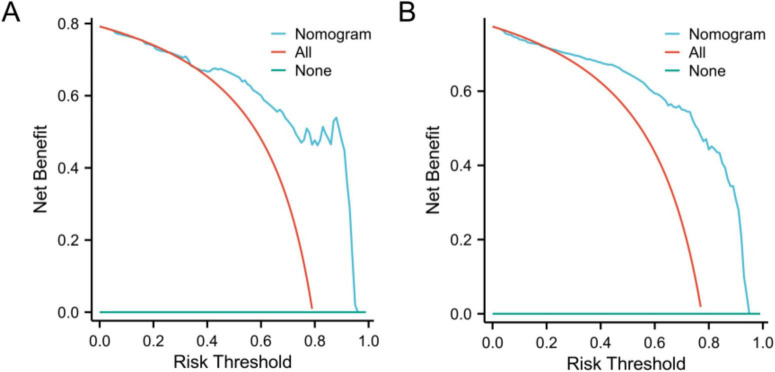
Decision curve analysis curves of the nomogram model in the training **(A)** and validation **(B)** cohorts.

The DCA curve was plotted to address the limitations of traditional evaluation indices, which can only measure the accuracy of the prediction model without considering the clinical utility of the specific model. The horizontal line represents no intervention or zero net income, and the oblique line represents intervention for all patients. The findings indicated that DCA demonstrated the ability of the model to predict sepsis in patients with liver abscesses. In both the training cohort (Figure 4A) and the validation cohort (Figure 4B).

## 4 Discussion

Liver abscess is an intrahepatic infectious disease with a notably high mortality rate. PLA remains a potentially life-threatening condition despite advancements in diagnostic and treatment approaches. Sepsis, a severe condition characterized by life-threatening organ dysfunction caused by a dysregulated host response to infection (5), has emerged as a major cause of mortality worldwide, with claimed millions of lives every year (10). Patients with liver abscesses who develop sepsis face a significantly increased risk of disease progression. Although risk factors associated with sepsis in patients with liver abscesses have been identified in previous studies, no nomogram integrating these factors has been developed to predict the likelihood of sepsis in this population. Sepsis is a severe complication of infectious diseases that substantially increases the risk of septic shock, leading to severe organ failure and potential mortality in patients with liver abscesses (11). Research has shown that delaying treatment significantly increases the mortality rate of sepsis patients (12). Therefore, an early identification of high-risk patients with liver abscesses who may develop sepsis is crucial. This study adopts the Sepsis-3 definition, in which sepsis is defined as acute organ dysfunction secondary to infection, quantified by a SOFA score ≥ 2 or an increase of ≥2 points from baseline. The diagnosis of sepsis in patients is determined by microbiological evidence such as blood culture, imaging examinations, and laboratory tests: elevated levels of CRP and dynamic changes in SOFA scores. Sepsis outcomes were assessed through serial SOFA score measurements to quantify organ dysfunction trends. Infection control efficacy was evaluated using inflammatory biomarkers, including CRP levels, with normalization trends indicating resolution of systemic inflammation.

Predictive models for sepsis risk assessment have been developed and validated in previous studies for patients with conditions such as urinary tract stones (13), postoperative gastric cancer (14), and pancreatitis (15). However, there is a lack of predictive models for sepsis in patients with liver abscesses. This retrospective cohort study analyzed cases over a 10-year period. Despite diagnostic advancements during this period, pathogen distribution patterns showed no significant temporal variation. Data were partitioned chronologically into training and validation sets, with temporal partitioning designed to align with clinical implementation requirements. This approach ensures that the prediction model’s validation reflects its anticipated real-world performance when applied prospectively to future patient cohorts. Consequently, in this study, a model using four clinical indicators (WBC, INR, gas formation, and SOFA score) was developed to predict the probability of sepsis in patients with liver abscesses.

White blood cell count is a commonly employed inflammatory indicator routinely measured upon admission. It is a convenient tool for early sepsis detection. However, it is often used in combination with other indicators for sepsis diagnosis because of its low specificity. Following bacterial invasion, serum lipopolysaccharide-binding protein (LBP) captures lipopolysaccharide (LPS) and transfers it to CD14 receptors on macrophages. CD14 facilitates LPS delivery to the Toll-like receptor 4 (TLR4)-myeloid differentiation factor 2 (MD-2) complex through direct molecular interactions, thereby activating two different signaling pathways: the MyD88-dependent and MyD88-independent pathways. These signaling events drive macrophage polarization toward the pro-inflammatory M1 phenotype, stimulate the secretion of inflammatory cytokines (e.g., TNF-α and IL-6), promote bone marrow release of immature neutrophils, and ultimately elevate peripheral blood leukocyte counts (16). Studies have demonstrated that normal WBC counts and mean distribution width counts can help exclude the diagnosis of sepsis (17). The early diagnosis of sepsis can be facilitated by a decrease and significant increase in serum WBC count in patients with bacterial infection (18). Our results are consistent with those of previous studies, further highlighting the significance of WBC count in evaluating sepsis risk in patients with liver abscesses. Thus, close attention and comprehensive evaluation are recommended for patients with significant changes in WBC count.

The computation of INR is based on prothrombin time and the international sensitivity index of the reagent, enabling the comparability of findings obtained using various thrombin reagents. It is commonly employed to assess coagulation time and monitor the effectiveness of anticoagulant drugs. Sepsis-induced coagulopathy (SIC) arises through a synergistic interplay of inflammatory, vascular, and microbial mechanisms. Proinflammatory cytokines such as IL-6 and TNF-α induce endothelial and monocytic overexpression of tissue factor (TF), activating the extrinsic coagulation pathway through thrombin generation, which drives fibrin deposition and microvascular thrombosis. Concurrently, cytokine-mediated downregulation of endothelial thrombomodulin (TM) suppresses activated protein C (APC) production, crippling endogenous anticoagulant defenses (19, 20). Bacterial virulence factors exacerbate this imbalance: endotoxins directly activate factor XII to initiate the intrinsic coagulation cascade while promoting platelet hyperaggregation, whereas exotoxins inflict endothelial injury through collagen exposure and protease activity, creating a proadhesive surface for circulating platelets. These intersecting pathways culminate in disseminated intravascular coagulation (DIC), characterized by paradoxical microthrombosis with coagulant consumption, ultimately manifesting as elevated INR and multi-organ perfusion failure. A strong association between INR and the diagnosis and prognosis of sepsis has been demonstrated in recent studies. Liu et al. identified INR as a crucial indicator of sepsis diagnosis (21). According to Sreeraj (22), in patients with sepsis, the ratio of INR to albumin represents an independent risk factor for mortality and can be employed for the early evaluation of disease severity and progression. In addition, a higher INR value at the time of diagnosis signifies a greater severity of organ failure (23). Our results indicate that patients with sepsis typically exhibit higher INR levels than those without sepsis, which could function as a reliable indicator for predicting sepsis in patients with liver abscesses, consistent with previous studies. Sepsis can result in coagulation disorders in the body, which are associated with disease severity, playing a critical role in the progression of organ dysfunction (24). Accurate recognition of the coagulation status of a patient, early attention, and enhanced monitoring of INR levels in patients with liver abscesses prove advantageous in guiding treatment and reducing the likelihood of poor prognosis.

Gas-forming PLA (GFPLA), which is characterized by gas within an abscess, is considered a rare and potentially life-threatening condition (25). The accumulation of gas causes local tissue damage and abscess formation, hindering the transport of gas and nutrients (2). GFPLA patients exhibit more severe infection, a higher incidence of sepsis, and a longer duration of hospital stay than non-GFPLA patients (26). Within the hepatic abscess microenvironment, pathogenic bacteria engage in mixed acid fermentation, generating formic acid as a metabolic byproduct. Under acidic conditions (pH < 6), bacterial formate hydrogenlyase catalyzes the conversion of formic acid to carbon dioxide (CO_2_) and hydrogen gas (H_2_) (27). This gas accumulation elevates intracavitary pressure within the abscess, which may predispose GFPLA cases to systemic bacteremia and spontaneous rupture. Immunocompromised patients frequently experience rapid clinical deterioration, progressing to sepsis due to impaired pathogen containment mechanisms (25). In addition, Wang et al. (28) established a close relationship between gas formation and abscess rupture, with severe cases presenting a life-threatening risk. Therefore, the risk of sepsis in these patients can be significantly reduced through the early identification of gas-containing liver abscesses and the timely implementation of specific and effective treatment measures. According to Etinosa and Janell (29), ultrasound is a highly accurate imaging method for diagnosing gas-containing abscesses and can function as a rapid screening tool, potentially replacing the necessity for CT examination. Nevertheless, this requires clinicians to have a thorough comprehension of the imaging results related to gas-bearing abscesses. GFPLA demonstrates distinct multimodality imaging features. On ultrasonography, intralesional gas appears as scattered, linear, or clustered hyperechoic foci. This phenomenon arises from repeated sound wave reflection at gas–tissue interfaces, producing multiple linear reverberation artifacts posterior to the gas collections (termed the “ring-down” or “comet-tail” sign). CT reveals hypoattenuating gas pockets with Hounsfield units (HU) approximating −1,000, markedly distinct from both purulent material (−10 to 30 HU) and hepatic parenchyma (40–70 HU). Magnetic resonance imaging exhibits signal voids on both T1- and T2-weighted sequences due to gas-induced magnetic susceptibility artifacts, which are accentuated on gradient-echo sequences. While no formal diagnostic criteria exist for GFPLA, their characteristic ultrasonographic, CT, and MR features enable conclusive radiographic identification (30). Our study indicates that the presence of gas in liver abscesses holds substantial predictive value for assessing sepsis risk and is considered a crucial factor in early sepsis screening.

The updated Sepsis-3 criteria have clarified clinical understanding of this condition by incorporating acute organ dysfunction into its diagnostic framework, reflecting the intricate interplay between systemic infection and pathological host responses. The SOFA score has been established as the diagnostic criterion for sepsis due to its validated capacity to objectively quantify multiorgan dysfunction severity and track disease progression (31). SOFA is a scoring system employed for assessing and predicting the condition and prognosis of septic patients. It has been extensively used in clinical practice (32). In the context of infection, sepsis is defined as a 2-point increase in the SOFA score compared with the baseline value (33), rendering it an effective predictive tool for sepsis. Our results reconfirm the independent risk factor status of SOFA in the development of sepsis among patients with liver abscesses. However, some data may not be available upon initial admission because of the involvement of multiple organs, potentially resulting in delays in diagnosis, inadequate sensitivity, and complex evaluation. Therefore, the SOFA score of patients with liver abscesses must be promptly evaluated upon admission by doctors, and the inclusion of relevant examination parameters in the scoring system should be actively enhanced. Timely intervention and implementation of effective treatment measures are necessary if there is a change of more than 2 points in the SOFA score within a short period or a noticeable upward trend.

While previous studies have identified CRP, PLT, and abscess size exceeding 5 cm as potential predictors of sepsis (34), these variables failed to demonstrate independent prognostic significance in our cohort. This discrepancy may be attributed to several factors. CRP, though indicative of systemic inflammation, exhibits limited specificity in distinguishing infection sources (e.g., bacterial vs. parasitic etiology) or disease progression stages in PLA patients. Furthermore, CRP levels are confounded by comorbidities (e.g., diabetes mellitus) and therapeutic interventions (e.g., antibiotic administration), thereby weakening its causal association with sepsis. Regarding platelet dynamics, concurrent DIC and pre-existing cirrhosis may drive thrombocytopenia, while bacterial endotoxin-mediated activation of mononuclear phagocyte pathways induces compensatory thrombocytosis, collectively obscuring sepsis-related depletion patterns. Although enlarged abscess dimensions (>5 cm diameter) were historically considered high-risk, our data suggest a non-linear threshold effect between infectious burden and sepsis likelihood. Additionally, volumetric measurement inaccuracies in multiloculated abscesses may attenuate statistical associations.

The model demonstrated robust discriminatory performance, with AUC values of 0.880 in the training set and 0.901 in the test set. Although the Hosmer–Lemeshow test indicated no significant overall calibration issues (χ^2^ = 8.60, *P* = 0.377), graphical analysis of calibration curves revealed systematic bias patterns. Specifically, the model demonstrated slight underestimation in both high- and low-risk subgroups, whereas symmetrical overestimation was observed in the intermediate-risk subgroup. This bidirectional miscalibration likely stems from overrepresentation of intermediate-risk patients in the training cohort, leading to overfitting to this subgroup, coupled with inadequate characterization of extreme risk values. Despite lacking statistical significance, these discrepancies carry clinical implications: persistent underestimation in high-risk patients could delay critical interventions (e.g., ICU transfers), while optimistic low-risk predictions might postpone necessary surveillance. Furthermore, the substantial intermediate-risk subgroup may experience unnecessary resource utilization due to systematic overestimation. Future directions include implementing adversarial training to enhance subgroup representativeness and developing risk-stratified recalibration algorithms to independently optimize prediction accuracy across risk tiers. DCA demonstrated that the nomogram provided superior clinical utility when the risk probability threshold exceeded 20%. At this threshold, a total score of 36 points on the nomogram scale was identified as the critical value for clinical intervention. Patients scoring above 36 points would benefit from therapeutic escalation through either antibiotic regimen intensification or percutaneous drainage, thereby optimizing resource allocation while maintaining therapeutic efficacy.

However, this study has some limitations. (1) This study is a retrospective analysis, making it susceptible to selective bias that cannot be disregarded. Heterogeneity in sepsis onset-to-sampling intervals across the study population may confound inflammatory biomarker measurements due to unstandardized sample collection timing. This variability could influence observed inflammatory states, as CRP levels fluctuate dynamically during sepsis progression. (2) Some crucial parameters, including procalcitonin, interleukin, and lactic acid, are often not evaluated or significantly missing upon admission, resulting in their exclusion in this study. Subsequent investigations should further explore the potential impact of these indicators on sepsis. (3) All data were obtained from a single center. Despite using different periods of patient samples to validate the model, further assessments and validations should be performed using data from other centers to establish robust evidence.

## 5 Conclusion

In summary, a predictive model that effectively predicts sepsis in patients with liver abscesses was developed. During validation, this model demonstrated good prediction and discrimination abilities. A nomogram that includes independent risk factors such as WBC, INR, gas formation, and SOFA score was introduced to simplify predictions for clinicians and enhance convenience. The nomogram demonstrates significant sensitivity and specificity and can be widely employed. It can assist clinicians in making personalized treatment and management decisions for patients with liver abscesses, consequently reducing the risk of sepsis and mortality.

## Data Availability

The raw data supporting the conclusions of this article will be made available by the authors, without undue reservation.
